# Glomerular capillary C3 deposition as a risk factor for unfavorable renal outcome in pediatric primary focal segmental glomerular sclerosis

**DOI:** 10.3389/fped.2023.1137375

**Published:** 2023-03-21

**Authors:** Yingchao Peng, Banghai Li, Xiaojie Li, Tao Ju, Zhiqiang Zhang, Peipei Wang, Tao Sun, Jiaping Shu, Meiqiu Wang, Xiaoyi Sun, Huangyu Chen, Chunlin Gao, Zhengkun Xia

**Affiliations:** ^1^ Department of Pediatrics, Affiliated Jinling Hospital, Medical School, Nanjing University, Nanjing, China; ^2^ Department of Medical Insurance Section, Zhujiang Hospital of Southern Medical University, Guangzhou, China; ^3^ Department of Pediatrics, Jinling Hospital, Nanjing Medical University, Nanjing, China; ^4^ Department of Medical Information, Jinling Hospital, Nanjing, China; ^5^ Department of Pediatrics, Jinling Hospital, Nanjing, China

**Keywords:** focal segmental glomerular sclerosis, capillary c3 deposition, renal outcome, risk factor, children

## Abstract

**Introduction:**

Some patients with primary focal segmental sclerosis (FSGS) demonstrate complement 3 (C3) deposition in glomerular capillary loops (Cap-C3) and/or mesangial area (Mes-C3). The clinicopathological and prognostic significance of C3 deposition remains incompletely investigated, especially in the pediatric cohort.

**Methods:**

We retrospectively analyzed 264 children of biopsy-proven primary FSGS between January 2003 and December 2020. The correlation between Cap-C3 and renal outcome was evaluated by the Kaplan-Meier method and Cox multivariate regression analysis. Renal end-point event was defined as the development of end-stage renal disease, death for renal disease, or an estimated glomerular filtration rate reduction by at least 50% from baseline.

**Results:**

Among the 264 patients, 30 (11.4%) had Cap-C3. Kaplan-Meier analysis showed that patients with Cap-C3 had significantly lower renal survival rates than patients without Cap-C3 (60.17% vs. 84.71% at 5 years, 39.49% vs. 65.55% at 10 years, *P* < 0.01). Cox multivariate regression analysis showed that Cap-C3 was an independent risk factor for poor renal outcome (HR 3.53, 95% CI 1.22–10.19, *P* = 0.02).

**Conclusion:**

Glomerular capillary C3 deposition was an independent risk factor for unfavorable renal outcome in children with primary FSGS.

## Introduction

Focal segmental glomerulosclerosis (FSGS), which accounts for approximately 20% of cases with pediatric nephrotic syndrome (NS), is one of the most common primary glomerular disorders causing childhood end-stage renal disease (ESRD) (1). The primary FSGS is clinical and pathologically heterogeneous and displays various renal outcomes, emphasizing the need to identify prognosis. Previous studies have identified some risk factors for poor renal prognosis, including reduced renal function, interstitial fibrosis, glomerular sclerosis, hypertension, and heavy proteinuria (2). Several studies have indicated that complement activation was presented in experimental animal models with FSGS and FSGS patients (3–6). Complement 3 (C3), a crucial material in three complement pathways, could deposit in the glomerulus of a subset of patients with FSGS (7). It is usually located in glomerular vascular loops, sometimes only in mesangial areas. However, the clinical and prognostic significance of glomerular C3 deposition remains unclear. Moreover, glomerular C3 deposition and its’ location have not been evaluated in any other large cohort, especially in the children cohort. This retrospective study first assessed the distribution of glomerular C3 deposition and its predictive value in progression in 264 Chinese children with primary FSGS.

## Methods

### Patients

**Figure 1:**
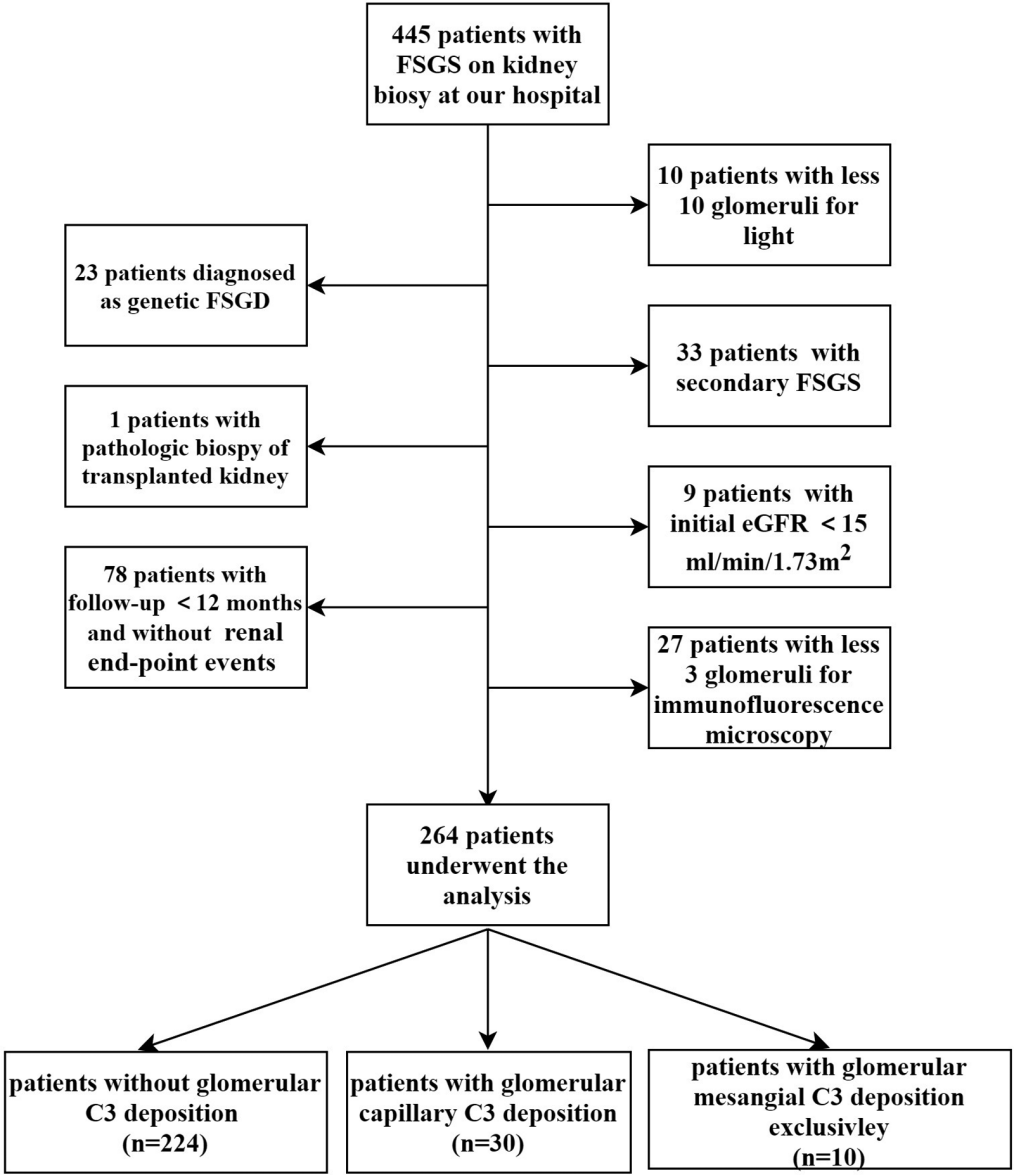
Flow chart of study enrollment.

Children (≤18 years) with primary FSGS diagnosed by kidney biopsy from January 2003 to December 2020 were recruited from Jinling hospital. The indication for kidney biopsy in this cohort was as follows: ① Children who were first diagnosed with NS at the age of ≥12 years old; ② Children with steroid-resistant NS (SRNS); ③ Children with atypical features, including macroscopic hematuria, hypertension, acute kidney injury no related to hypovolemia, rash suggesting glomerulonephritis; ④ Steroid-sensitive NS (SSNS) patients progress to secondary SRNS, steroid-dependent NS or frequent relapsing NS in the course of follow-up treatment. We excluded the patients with transplanted kidneys and secondary FSGS, such as obesity-induced FSGS, virus-associated FSGS, drug-induced FSGS, genetic FSGS, and segmental sclerosis lesions complicated with other glomerular diseases. Exclusion criteria in this study were initial estimated glomerular filtration rate (eGFR) < 15 ml/min/1.73 m^2^, the glomerular number on light microscopy <10, or the glomerular number on immunofluorescence microscopy <3. In addition, cases with follow-up <12 months were excluded, except those who progressed to the renal end-point event within 12 months. Finally, 264 patients were enrolled in this single-center retrospective study (Figure 1).

### Clinical and pathological data

All clinical data were obtained retrospectively from medical records. The baseline investigations were taken at the renal biopsy, and follow-up was conducted through December 2021. The eGFR was estimated by the Schwartz formula (≤16 years) (8) or the CKD-EPI equation (>16 years) (9). The diagnosis of hypertension was based on the 2017 AAP Blood Pressure Clinical Practice Guidelines (10). Nephrotic-range proteinuria and NS were defined according to the IPNA definitions (11).

All patients underwent renal biopsy at the time of diagnosis. Renal specimens were evaluated with light microscopy, direct immunofluorescence, and electron microscopy. The Immunofluorescence staining of immunoglobulin G (IgG), immunoglobulin A (IgA), immunoglobulin M (IgM), complement 1q (C1q), complement 4 (C4), and C3 was examined. Immunofluorescence staining positive was defined as the intensity of the staining by immunofluorescence ≥1+. The locations of C3 and IgM were also evaluated. “Cap-C3” was defined as glomerular capillary C3. “Mes-C3 exclusively” was defined as C3 exclusively staining in glomerular mesangial areas. Histologic variants were determined according to the Columbia classification (12). The change of chronic tubulointerstitial injury (CTI) indicated tubular atrophy/interstitial fibrosis.

### Evaluation of treatment response and renal outcome

(1) Steroid-sensitive was defined as complete remission (CR) within 4 weeks of prednisone or prednisolone (PDN) at the standard dose (60 mg/m^2^/day or 2 mg/kg/day, maximum 60 mg/day) (11). (2) Steroid-dependent was defined as follows: ① Two consecutive relapses during steroids therapy or within 15 days of discontinuing steroids therapy; ② Infectious factors should be excluded at the same time; ③ Once increased the dose of steroids (either at full dose or a dose more than the recurrence dose), patients could reach CR. (3) Initial steroid-resistant was defined as lack of CR within 4 weeks of treatment with steroids at standard dose (11). (4) Secondary steroid-resistant was defined as children with initial SSNS who developed SRNS after subsequent relapses (11). (5) Partial remission (PR) was defined as urine protein/creatinine ratio (UPCR) (based on first morning void or daily urine sample) >20 but <200 mg/mmol and, if available, serum albumin ≥30 g/L (11). (6) CR was defined as UPCR (based on first morning void or daily urine sample) ≤20 mg/mmol (0.2 mg/mg) or negative or trace dipstick on three or more consecutive occasions (11). (7) Remission was defined as the achievement of PR or CR. (8) Renal end-point event was defined as the development of ESRD, death for renal disease, or an eGFR reduction by at least 50% from baseline. (9) ESRD was defined as two consecutive eGFR < 15 ml/min/1.73 m^2^ within a month, or kidney transplant, or dialysis duration ≥ 3 months. (10) Renal survival time was defined as when cases reached the renal end-point or the last follow-up if patients did not enter the end-point event during the entire follow-up visit.

### Statistical analysis

SPSS (version 26.0) and GraphPad Prism 8 were used for statistical analysis. Continuous variables were presented as mean ± SD deviation or median (interquartile range, Q1–Q3) and analyzed with ANOVA or Kruskal-Wallis *H* test. Categorical data were expressed as frequency (%) and analyzed using the Chi-square test or Fischer's exact test. Kidney survival rates were calculated using the Kaplan-Meier method. Parameters with a *P*-value <0.05 in the Cox univariate analysis were recognized as confounding factors and were included in the multivariate Cox proportional hazard model. “Enter” methods were used to identify the independent prognostic indicators among the confounding variables. Results were expressed as hazard ratios (HRs) and 95% confidence intervals (95% CIs). A two-tailed value of *P*-value <0.05 was considered statistically significant.

## Results

### Distribution of depositions by direct immunofluorescence

The distribution of depositions by direct immunofluorescence is shown in Table 1. The occurrences of different deposits by direct immunofluorescence were as follows: 4.5% with IgG, 14.0% with IgA, 41.3% with IgM, 15.2% with C3, 9.1% with C1q, 1.9% with C4, 20.8% with Cap-IgM, 11.4% with Cap-C3 and 3.8% with Cap-C1q. According to the end-point event, the cohort was divided into two groups: No end-point group and End-point group. There were no significant differences in the occurrence of IgG (2.1% vs. 5.1%, *P* = 0.70), IgA (16.7% vs. 13.4%, *P* = 0.65), IgM (56.3% vs. 40.7%, *P* = 0.70), C1q (6.3% vs. 9.7%, *P* = 0.59) and C4 (1.9% vs. 2.1%, *P* = 1.00) between patients with and without end-point events. The End-point group had a significantly higher occurrence of C3 deposition than the No end-point group (25.0% vs. 13.0%, *P* = 0.04). When focusing on the deposition in glomerular capillary loops, the End-point group had a significantly higher occurrence of Cap-C3 (25.0% vs. 8.3%, *P* = 0.001) and Cap-IgM (33.3% vs. 18.1%, *P* = 0.02) than the No end-point group. However, no significant difference in Cap-C1q was detected between the two groups.

**Table 1:** Renal immunofluorescence microscopy features of pediatric FSGS.

Items	Total	No end-point	End-point	*P*
	*n* = 264 (100.0%)	*n* = 216 (81.8%)	*n* = 48 (18.2%)	
IgG+, *n* (%)	12 (4.5)	11 (5.1)	1 (2.1)	0.70
IgA+, *n* (%)	37 (14.0)	29 (13.4)	8 (16.7)	0.65
IgM+, *n* (%)	109 (41.3)	88 (40.7)	27 (56.3)	0.70
C3+, *n* (%)	40 (15.2)	28 (13.0)	12 (25.0)	0.04
C1q+, *n* (%)	24 (9.1)	21 (9.7)	3 (6.3)	0.59
C4+, *n* (%)	5 (1.9)	1 (2.1)	4 (1.9)	1.00
Cap-IgM+, *n* (%)	55 (20.8)	39 (18.1)	16 (33.3)	0.02
Cap-C3+, *n* (%)	30 (11.4)	18 (8.3)	12 (25.0)	<0.01
Cap-C1q+, *n* (%)	10 (3.8)	8 (3.7)	2 (4.2)	1.00

### Other baseline clinicopathologic data

200 (75.8%) patients were males, and 64 (24.2%) were females. At biopsy, the median age was 15.4 (13.1–17.0) years; the median proteinuria was 6.1 (2.7–10.2) g/day; the median eGFR was 132.2 (87.0–162.3) ml/min/1.73 m^2^. The number of patients with NS was 160 (60.6%), with hypertension was 114 (43.3%), and with hematuria was 182 (68.9%). 123 (46.6%), 80 (30.3%), 27 (10.2%), 27 (10.2%) and 7 (2.7%) patients were diagnosed with NOS, Tip, Collapsing, Cellular and Perihilar, respectively. Based on C3 deposition and its’ location, the cohort was divided into three groups as follows: “C3− group”, “Cap-C3 + group”, “Mes-C3 + exclusively group”, which amounted to 224 (84.8%), 30 (11.4%) and 10 (3.8%), respectively. Comparisons of the baseline clinicopathologic characteristics among the groups are listed in Table 2.

**Table 2:** Baseline demographic and clinicopathologic characteristics of pediatric FSGS with and without glomerular C3 deposition.

Items	C3−	Cap-C3+	Mes-C3 + exclusively	*P*
	*n* = 224 (84.8%)	*n* = 30 (11.4%)	*n* = 10 (3.8%)	
Male, *n* (%)	171 (76.3)	21 (70.0)	8 (80.0)	0.71
Age at biopsy, years	15.3 (13.1–17.0)	15.5 (13.1–17.0)	16.0 (12.3–18.0)	0.75
Duration, months	3.0 (1.0–18.5)	6.0 (1.0–30.0)	1.0 (0.8–5.0)	0.08
Hypertension, *n* (%)	98 (43.8)	10 (33.3)	6 (60.0)	0.31
NS, *n* (%)	134 (59.8)	21 (70.0)	5 (50.0)	0.44
Serum album, g/L	21.0 (18.0–27.5)	20.7 (18.4–25.1)	23.5 (20.0–27.0)	0.42
eGFR, ml/min/1.73 m^2^	132.4 (87.0–162.9)	131.7 (78.0–149.1)	129.0 (103.5–220.4)	0.54
Serum IgG, g/L	2.8 (1.8–4.4)**	1.8 (1.5–3.0)*	2.6 (2.1–3.6)	0.04
Serum IgA, g/L	1.6 (1.2–2.0)**	1.3 (1.0–1.5)*	1.3 (1.2–1.9)	0.01
Serum IgM, g/L	1.6 (1.1–2.1)	1.6 (1.4–2.1)	1.7 (1.5–2.1)	0.42
Serum C3, g/L	1.0 (0.9–1.2)	1.0 (0.8–1.1)	1.0 (0.8–1.1)	0.16
Serum C4, g/L	0.2 (0.2–0.3)**	0.3 (0.2–0.3)	0.2 (0.2–0.3)	0.58
Hematuria, *n* (%)	153 (68.3)	24 (80.0)	5 (50.0)	0.18
Urinary protein, g/day	6.0 (2.7–9.9)	7.0 (3.9–10.6)	4.7 (1.6–11.2)	0.56
Global sclerosis, %	0.0 (0.0–2.7)**	3.8 (0.0–20.8)*	0.0 (0.0–0.8)**	<0.01
Segmental sclerosis, %	11.5 (6.1–20.0)	18.6 (5.7–31.3)	11.6 (5.2–19.4)	0.22
CTI <25%	210 (93.8)**	24 (80.0)*	10 (100.0)*, **	0.04
CTI ≥25%	14 (6.3)**	6 (20.0)*	0 (0.0)*, **	
IgG+, *n* (%)	3 (1.3)**	6 (20.0)*	3 (30.0)*	<0.01
IgA+, *n* (%)	27 (12.1)**	6 (20.0)*	4 (40.0)*, **	0.03
IgM+, *n* (%)	79 (35.3)**	22 (73.3)*	8 (80.0)*	<0.01
C4+, *n* (%)	0 (0.0)**	5 (16.7)*	0 (0.0)**	<0.01
C1q+, *n* (%)	12 (5.4)**	9 (30.0)*	3 (30.0)*	<0.01
Intensity of C3 deposition				
1+	-	0 (0.0)	4 (40.0)**	<0.01
2+	-	14 (46.7)	5 (50.0)	
3+	-	16 (53.3)	1 (10.0)**	
**Variants**				
Nos	100 (44.6)	16 (53.3)	7 (70.0)	0.64
Tip	70 (31.3)	8 (26.7)	2 (20.0)	
Collapsing	24 (10.7)	3 (10.0)	0 (0.0)	
Cellular	24 (10.7)	3 (10.0)	0 (0.0)	
Perihilar	6 (2.7)	0 (0.0)	1 (10.0)	

NS, nephrotic syndrome; eGFR, estimated glomerular filtration rate; CTI, chronic tubulointerstitial injury; Nos, not otherwise specified.

* *P* < 0.05 vs. C3−.

** *P* < 0.05 vs. Cap-C3+.

There were no statistically significant differences in the following demographic and clinical parameters among the three groups: sex, age at biopsy, duration, the proportion of patients with hypertension, the proportion of patients with NS, serum album, eGFR, serum IgM, serum C3, serum C4, the proportion of patients with hematuria, and daily urinary protein. The Cap-C3 + group had a significantly lower level of serum IgG than the C3− group and the Mes-C3 + exclusively group (median, IQR: 1.8, 1.5–3.0 g/L vs. 2.8, 1.8–4.4 g/L and 2.6, 2.1–3.6 g/L, respectively, *P* = 0.04). And the Cap-C3 + group had a significantly lower level of serum IgA compared with the C3− group and the Mes-C3 + exclusively group (median, IQR: 1.3, 1.0–1.5 g/L vs. 1.6, 1.2–2.0 g/L and 1.3, 1.2–1.9 g/L, respectively, *P* = 0.01).

There was no statistically significant difference in glomerular segmental sclerosis and variants among the three groups. The Cap-C3 + group had a significantly higher percentage of patients with more than 25% of CTI compared to the C3− group and the Mes-C3 + exclusively group (20.0% vs. 6.3% and 0.0%, respectively, *P* = 0.04). In addition, the Cap-C3 + group had a significantly higher proportion of patients with 3 + intensity of C3 deposition than the Mes-C3 + exclusively group (53.3% vs. 10.0%, *P* < 0.01). Other significant deposition parameters among groups included IgG, IgA, IgM, C4 and C1q. There was no significant difference in IgM deposition between the Cap-C3 + group and the Mes-C3 + exclusively group (73.3% vs. 80.0%, *P* > 0.05). However, the C3− group had a significantly lower proportion of patients with IgM deposition than the Cap-C3 + group and the Mes-C3 + exclusively group (35.3% vs. 73.3% and 80.0%, respectively, *P* < 0.01). Likewise, no significant difference in C1q deposition was detected between the Cap-C3 + group and the Mes-C3 + exclusively group (30.0% vs. 30.0%, *P* > 0.05). But the C3− group had a significantly lower proportion of patients with C1q deposition than the Cap-C3 + group and the Mes-C3 + exclusively group (5.4% vs. 30.0% and 30.0%, respectively, *P* < 0.01).

### Treatment

Cases with sub-nephrotic proteinuria were initially managed with angiotensin-converting enzyme inhibitors (ACEI) or angiotensin receptor blockers (ARB). And immunosuppressive agents (IA) were also administered when these patients’ kidney function and proteinuria worsened. During a median observation period of 39.9 (18.4–62.3) months, 264 (100%) patients received steroids due to nephrotic-range proteinuria, progression of renal function, or worsening proteinuria. As shown in Table 3, no statistical differences in treatment and response to steroids among the groups were observed.

**Table 3:** Treatment, therapeutic response and renal outcome of pediatric FSGS with and without glomerular C3 deposition.

Items	C3−	Cap-C3+	Mes-C3 + exclusively	*P*
	*n* = 224 (84.8%)	*n* = 30 (11.4%)	*n* = 10 (3.8%)	
**Follow-up, months**	41.5 (19.4–61.7)	24.8 (12.3–67.0)	54.0 (23.6–74.7)	0.19
**ACEI/ARB**	124 (55.4)	14 (46.7)	6 (60.0)	0.63
**Steroids**	224 (100.0)	30 (100.0)	10 (100.0)	-
**IA**	205 (91.5)	29 (96.7)	10 (100.0)	0.66
TAC/CsA	89 (39.7)	12 (40.0)	5 (50.0)	0.81
MMF	11 (4.9)	2 (6.7)	1 (10.0)	0.44
LEF	126 (56.3)	19 (63.3)	7 (70.0)	0.55
TG	166 (74.1)	25 (83.3)	8 (80.0)	0.51
CTX	22 (9.8)	2 (6.7)	2 (20.0)	0.40
RTX	3 (1.3)	0 (0.0)	0 (0.0)	1.00
**Responses to steroids, *n* (%)**				
Steroid-sensitive (including steroid-dependent)	110 (49.1)	12 (40.0)	3 (30.0)	0.48
Initial steroid-resistant	95 (42.4)	15 (50.0)	7 (70.0)	
Secondary steroid-resistant	19 (8.5)	3 (10.0)	0 (0.0)	
**Treatment responses, *n* (%)**				
CR	41 (18.3)	2 (6.7)	2 (20.0)	<0.01
PR	124 (55.4)	10 (33.3)	7 (70.0)	
NR	59 (26.3)**	18 (60.0)*	1 (10.0)**	
**The end-point event, *n* (%)**	36 (16.1)**	12 (40.0)*	0 (0.0)*, **	<0.01

ACEI, angiotensin-converting enzyme inhibitors; ARB, angiotensin receptor blockers; IA, immunosuppressive agents other than steroids; TAC, tacrolimus; CsA, cyclosporin A; MMF, mycophenolate mofetil; LEF, leflunomide; TG, tripterygium glycosides; CTX, cyclophosphamide; RTX, rituximab; CR, complete remission; PR, partial remission; NR, no remission.

* *P* < 0.05 vs. C3−.

** *P* < 0.05 vs. Cap-C3+.

IA other than steroids, such as Tacrolimus (TAC) and cyclosporin A (CsA), mycophenolate mofetil (MMF), leflunomide (LEF), tripterygium glycosides (TG), cyclophosphamide (CTX) and rituximab (RTX) were initiated in steroid-resistant or steroid-dependent patients. Totally, 244 (92.4%) received IA other than steroids. Throughout the whole course of the disease, 57 (21.6%) patients only received one kind of IA, 130 (49.2%) received two types of IA, and 57 (21.6%) received at least three kinds of IA.

At the last follow-up, 187 (70.8%) patients achieved remission, including 143 (54.2%) patients achieving PR and 45 (17.0%) achieving CR. 58 (18.2%) patients reached the end-point event after a median follow-up of 27.5 (12.9–59.0) months. Among them, 22 (8.3%) patients, excluding patients reaching ESRD or dead, had a more than 50% reduction in basic eGFR after a median follow-up of 32.2 (20.3–66.8) months. 24 (9.1%) patients progressed to ESRD after a median follow-up of 17.4 (9.6–40.2) months, and 2 (0.8%) patients were dead for cardiogenic shock and pulmonary edema after a follow-up of 4.6 and 49.8 months, respectively.

### Therapeutic response and renal outcome

The details of therapeutic response and renal outcome among the C3-group, the Cap-C3 + group and the Mes-C3 + exclusively group are shown in Table 3. The Cap-C3 + group had a significantly higher percentage of patients who did not reach remission compared with the C3− group and the Mes-C3 + exclusively group (60.0% vs. 26.3% and 10.0%, respectively, *P* < 0.01). At the same time, more patients entered the end-point event in the Cap-C3 + group than in the C3− group and the Mes-C3 + exclusively group (40.0% vs. 16.1% and 0.0%, respectively, *P* < 0.01). As patients with Cap-C3 presented the worst prognosis while patients of the Mes-C3 + exclusively group had the best prognosis, we combined the C3− group and Mes-C3 + group into one group (the Cap-C3− group) and compared it with the Cap-C3 + group in the Kaplan-Meier and Cox regression analyses.

**Figure 2:**
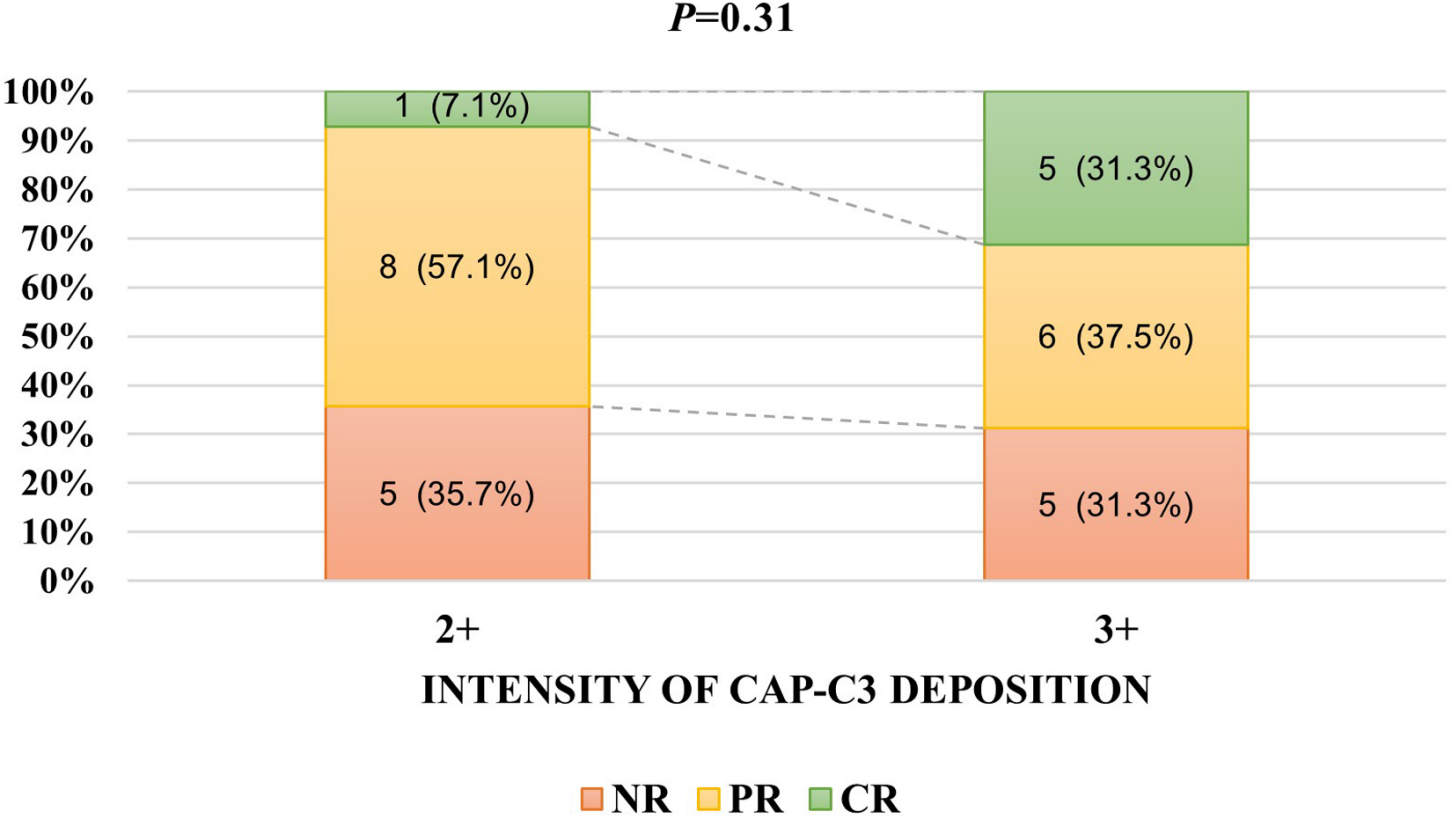
The relationship between the intensity of Cap-C3 deposition and responses to therapy.

**Figure 3:**
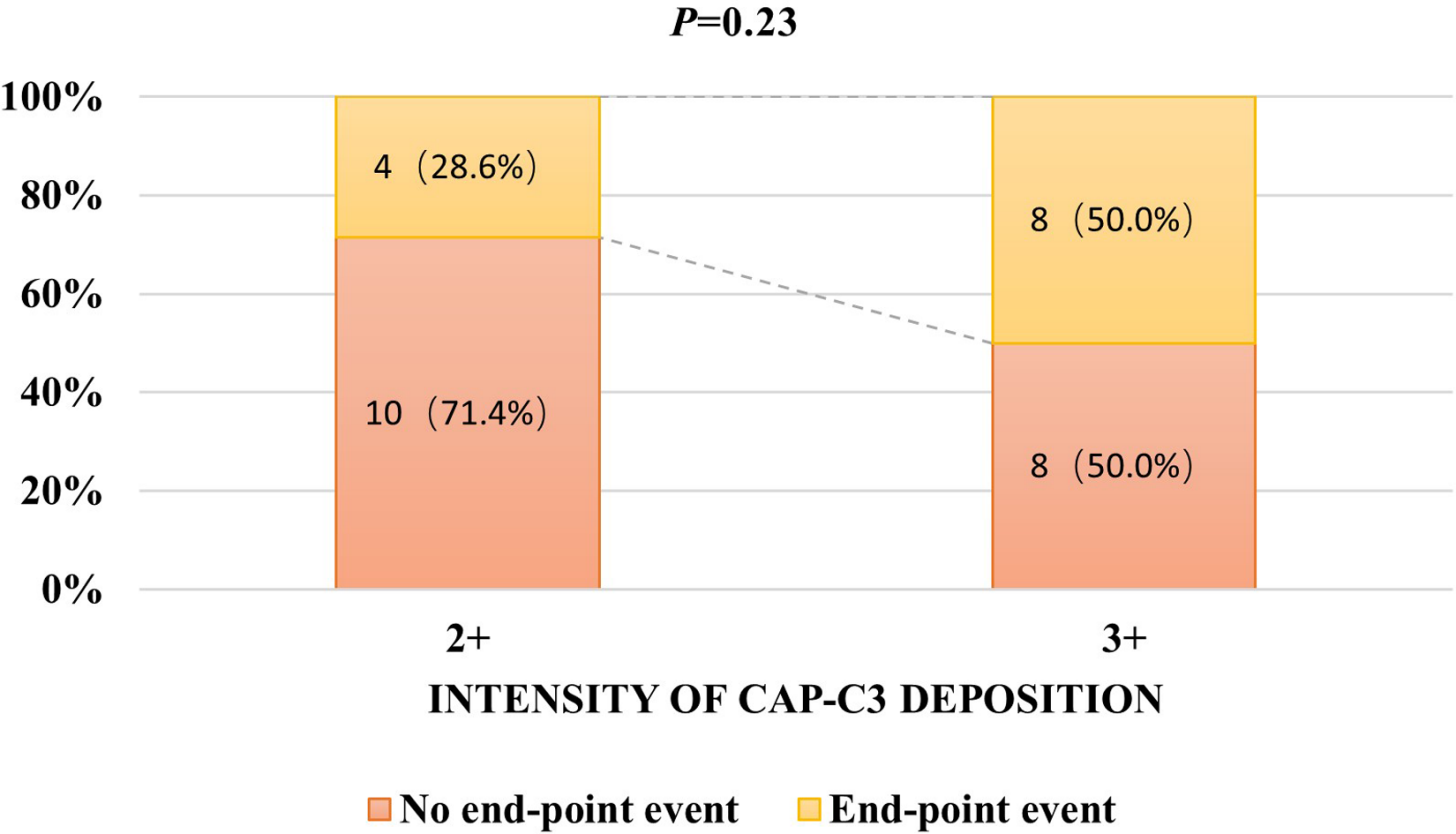
The relationship between the intensity of Cap-C3 deposition and end-point event.

The intensity of Cap-C3 was 2 + in 14 patients. At the same time, 16 patients had a 3 + intensity of Cap-C3. The intensity of Cap-C3 was unrelated to remission (*P* = 0.31; Figure 2). Moreover, the intensity of Cap-C3 was not associated with the end-point event (*P* = 0.23; Figure 3).

### Risk factors for unfavorable renal outcome

**Figure 4:**
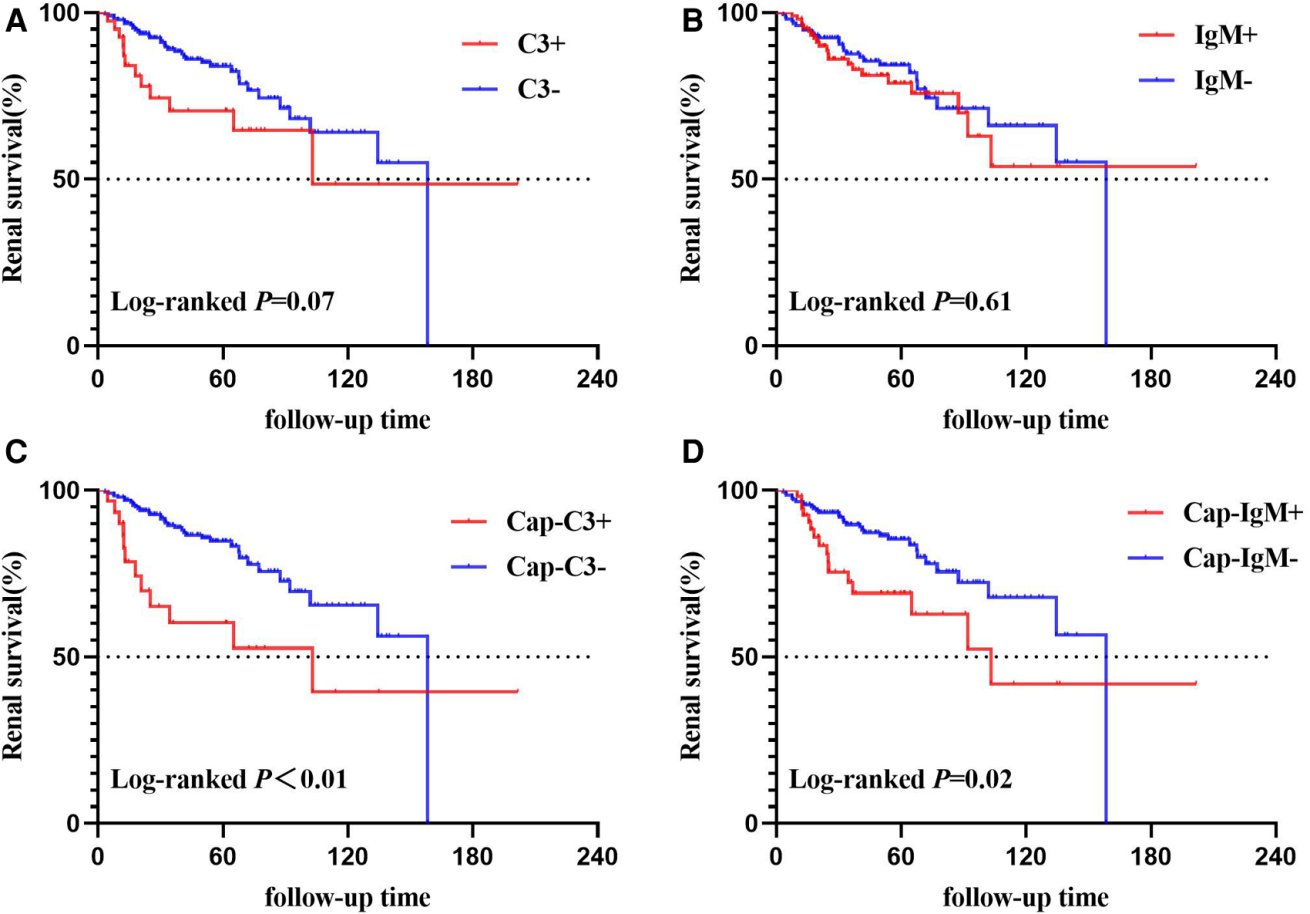
Kaplan–Meier curves of time to end-point events. (**A**) Based on C3 deposition; (**B**) based on IgM deposition; (**C**) based on Cap-C3 deposition; (**D**) based on Cap-IgM deposition.

The total renal survival rate of the end-point was 82.06% at 5 years and 61.62% at 10 years. The detailed results of the Kaplan-Meier survival analysis are shown in Figure 4. No significant difference in survival rates between patients with and without C3 was identified (*P* = 0.07; Figure 4A). Similarly, there was no statistically significant difference in survival rates between the patients with and without IgM deposition (*P* = 0.61; Figure 4B). When we focused on capillary deposition, the results were quite surprising. The survival rate of the Cap-C3 + group was significantly lower than the Cap-C3− group (*P* < 0.01; Figure 4C). The 5- and 10-year renal survival rates were 60.17% and 84.71%, 39.49% and 65.55% for the Cap-C3 + group and the Cap-C3− group, respectively. Likewise, the Cap-IgM + group had a significantly lower renal survival rate than the Cap-IgM− group (*P* = 0.02; Figure 4D). The 5- and 10-year renal survival rates were 69.12% and 85.26%, 41.89% and 67.87% for the Cap-IgM + group and the Cap-IgM− group, respectively. In a word, the results of the Kaplan-Meier survival analysis showed that the location of IgM and C3 was closely related to long-term renal survival. The findings were consistent with the results of the Cox analysis, which are presented in Table 4.

**Table 4:** Risk factors for renal outcome analyzed with Cox univariate and multivariate analyses.

Item	Cox univariate analysis		Cox multivariate analysis	
	HR (95% CI)	*P*	HR (95% CI)	*P*
Age at biopsy, years	0.92 (0.86–0.98)	<0.01	0.98 (0.90–1.07)	0.64
Sex, male	0.81 (0.41–1.64)	0.57	-	-
Hypertension	2.07 (1.15–3.72)	0.02	4.29 (1.99–9.24)	<0.01
NS	1.12 (0.62–2.02)	0.67	-	-
Proteinuria, g/day	1.00 (0.95–1.05)	0.93	-	-
Serum albumin, g/L	1.02 (0.99–1.05)	0.26	-	-
eGFR, ml/min/1.73 m^2^	1.00 (0.99–1.00)	0.07	-	-
Serum IgM, g/L	0.88 (0.60–1.28)	0.50	-	-
Serum C3, g/L	1.09 (0.45–2.64)	0.86	-	-
Global sclerosis, %	1.04 (1.03–1.05)	<0.01	1.01 (0.99–1.03)	0.30
Segmental sclerosis, %	1.04 (1.03–1.06)	<0.01	1.03 (1.01–1.05)	<0.01
CTI ≥25%	15.83 (8.54–29.35)	<0.01	8.39 (2.43–29.02)	<0.01
**Variants** [NOS (reference)]				
Tip	0.25 (0.10–0.67)	<0.01	0.45 (0.16–1.31)	0.14
Collapsing	3.25 (1.68–6.29)	<0.01	2.10 (1.01–4.40)	<0.05
Cellular	0.52 (0.16–1.75)	0.29	1.39 (0.38–5.06)	0.62
Perihilar	0.69 (0.16–2.99)	0.62	1.56 (0.34–7.28)	0.57
**C3+**	1.82 (0.94–3.52)	0.08	-	-
**IgM+**	1.16 (0.66–2.05)	0.61	-	-
**Cap-C3+**	2.54 (1.30–4.95)	<0.01	3.53 (1.22–10.19)	0.02
**Cap-IgM+**	2.02 (1.10–3.71)	0.02	0.78 (0.29–2.06)	0.61

NS, nephrotic syndrome; eGFR, estimated glomerular filtration rate; CTI, chronic tubulointerstitial injury; Nos, not otherwise specified.

The Cox univariate analysis showed that IgM deposition (HR = 1.16, 95% CI = 0.66–2.05, *P* = 0.61) and C3 deposition (HR = 1.82, 95% CI = 0.94–3.52, *P* = 0.08) did not reach statistical significance. But Cap-IgM deposition (HR = 2.02, 95% CI = 1.10–3.71, *P* = 0.02) and Cap-C3 deposition (HR = 2.54, 95% CI = 1.30–4.95, *P* < 0.01) were associated with the end-point event in the univariate analysis. Other significant parameters in the univariate analysis included age at biopsy, hypertension, global sclerosis, segmental sclerosis, CTI ≥25%, and variants. The Cox multivariate regression model revealed that Cap-C3 deposition was an independent risk factor for poor renal outcome (HR = 3.53, 95% CI = 1.22–10.19, *P* = 0.02) while Cap-IgM deposition was not correlated with renal outcome anymore (HR = 0.78, 95% CI = 0.29–2.06, *P* = 0.61). Other significant risk factors in the Cox multivariate analysis included hypertension, segmental sclerosis, CTI ≥25%, and variants. It should be explained that because all the patients in our study received a similar therapeutic principle and no significant differences in treatments were detected among groups (Table 3), treatment was not included as a potential confounding factor in the regression analysis.

## Discussion

This retrospective study assessed the distribution of glomerular depositions and correlated risk indicators in 264 Chinese children with primary FSGS. Currently, this study is the largest one in children with primary FSGS and first found that Cap-C3 deposition was an independent risk factor for deterioration of renal function after adjusting for confounders such as CTI, segmental sclerosis, and hypertension.

FSGS is never regarded as an immune complex-associated glomerulonephritis. But the role of complement in the non-immune injury of the kidney is not new, such as hypertensive nephropathy (13) and diabetic nephropathy (14). A human study showed that the plasma and urine levels of complement fragments in patients with FSGS were significantly higher than in control subjects (15). A study in mice showed that the factor D-deficient model was protected from renal disease caused by Adriamycin (4). These researches demonstrated that complement activation might play a crucial role in the pathogenesis and outcome of FSGS.

Over the past decades, many studies have assessed risk factors for ESRD in FSGS. Laboratory and clinicopathologic features, including decreased renal function, heavy proteinuria, hypertension, collapsing type, interstitial fibrosis, and global sclerosis, have been identified as independent risk factors for ESRD (2). However, few studies evaluated the role of complement activation in patients with FSGS. In recent years, several studies have begun researching the clinical significance of glomerular depositions in FSGS. Until now, there have been three articles concerning immunoglobulin and complement depositions to predict the prognosis of patients with primary FSGS (Table 5) (16–18).

**Table 5:** Studies about the prognostic significance of glomerular IgM/C3 deposition in primary FSGS.

Reference	Year	Country	Number	Age	Renal outcome	Location of IgM/C3	Independent risk factor
Zhang et al. (16)	2016	China	106	Adults	a need for renal replacement therapy or serum creatinine increasing >30% from the baseline and reaching >1.5 mg/dl	① Glomerulus ② C3 deposition was shown exclusively in patients with IgM deposition	IgM and C3 co-deposition
Pačić A et al. (17)	2017	Croatia	47	Adults	permanent increase in serum creatinine by ≥50% or ESRD, or a need for renal replacement therapy, or death	① Mesangial areas	None
Safak et al. (5)	2019	Turkey	86	Adults	≥50% reduction in baseline eGFR or eGFR <15 ml/min/1.73 m^2^	① Glomerulus ② C3 deposition was shown exclusively in patients with IgM deposition	IgM and C3 co-deposition
Present study	2022	China	264	Children	≥50% reduction in baseline eGFR, or two consecutive eGFR <15 ml/min/1.73 m^2^ within a month, or kidney transplant, or dialysis duration ≥3 months, or death for renal disease	① Capillary loops ② Mesangial areas	Capillary C3 deposition

ESRD end-stage renal disease; eGFR, estimated glomerular filtration rate.

C3 deposition may be closely related to IgM deposition in primary FSGS. Two studies found that IgM and C3 co-deposition, primarily deposited in sclerotic lesions, was significantly associated with poor renal prognosis in adults with primary FSGS (16, 18). But C3 deposition was shown exclusively in patients with IgM deposition in these two studies. It indicated that IgM might involve in renal injury by activating the complement system. Our study found that patients with C3 deposition more commonly show IgM deposition. Some scholars believed that IgM natural antibodies could bind to the *de novo* antigens exposed on the glomerulus, activate the complement system, and further contribute to renal injury (3, 19). And C3 deposition indicates an abnormal activation of the complement pathway.

The effect of the same deposition might differ in location. IgA nephropathy is characterized as glomerular mesangial IgA deposition. A recent study about IgA nephropathy showed that Cap-IgA deposition was associated with acute inflammation, glomerular basement membrane changes, and inferior prognosis (20). It is not uncommon that the clinical manifestation and renal progression are worse in patients with glomerular diseases and capillary deposition. For example, it is widely known that class III/IV lupus nephritis progresses faster than class I/II. Class III/IV lupus nephritis is characterized by subendothelial immune complex deposition, while class I/II is absent of subendothelial deposits. A study on adults focused on mesangial deposition rather than the deposition in sclerotic lesions, and its’ Cox multivariate analysis showed that mesangial IgM had no prognostic significance in primary FSGS (17). Up to now, no specific study has examined whether the location plays a crucial role in renal prognosis. Besides, clinical research investigating the role of deposition is limited to a few studies. Similar studies have not been carried out in the pediatric setting.

A majority of patients with C3 showed IgM deposition in this study. Thus, our study did not evaluate the prognostic significance of IgM and C3 co-deposition in the Cox multivariate analysis because C3 deposition interacted with co-deposition. Contrary to the adult findings, this study emphasized the importance of the location of C3 deposition. C3 deposition was regarded as a risk factor in adult primary FSGS but not in the children of our study (16, 18). Our results revealed that Cap-C3 rather than C3 was an independent risk factor for poor renal outcome. The result suggested that complement activation might be pathogenic in accelerating the progression of primary FSGS. Moreover, we suspected that the complement activation in glomerular capillary loops leads to more severe damage to the kidney. Or epitopes might be exposed to nearby glomerular loops because a recent study found that patients with FSGS have elevated levels of natural IgM reactive with epitopes on glomerular endothelial cells (21).

Morphologically, the continuous glomerular filtration membrane (GFM), composed of fenestrated glomerular endothelial cells, the glomerular basement membrane, and the podocytes, was along capillary loops. Cap-C3 deposition might represent the complement activation in GFM. FSGS is a quintessential podocyte disease. Podocytes can secrete vascular endothelial growth factor, endothelin-1, angiopoietin, and other substances to maintain the normal function of endothelial cells (22–24). Meanwhile, glomerular endothelial injury can also affect the operation of podocytes (25). Glomerular mesangial cells have a recovery function and can absorb and break down large immune molecules from the glomerular filtration membrane (GFM). We proposed that Mes-C3 deposition may represent a mild complement activation that serves as a repair process to remove the renal apoptotic cells. Cap-C3 deposition may indicate that GFM is hitting by ongoing inflammation caused by the complement activation, which is beyond compensatory capacity and results in local tissue damage.

The possible reason for the discrepant results between adults and children is that Cap-C3 deposition may be more frequent in adult FSGS. The study by Zhang et al. found that 18.87% (20/106) of adults with primary FSGS had C3 deposition on sclerotic segments (16). In comparison, our study had a lower frequency of Cap-C3, which accounted for 11.40%. The C3 deposition on sclerotic segments and those in the mesangium of unaffected areas were defined as C3 deposition in the study by Safak et al. (18). Thus, it is impossible to compare the frequency of Cap-C3 deposition between our research and the study by Safak et al. (18). In a word, a clinical study comparing the frequency of Cap-C3 between adults and children, needs to be performed to further confirmed the speculation, because no study based on adult cohort have analyzed the location of C3. Panzer et al. found that complement-mediated injury to the kidney was related to immunoglobulin deposition in capillary walls as the mice aged (19). A higher frequency of Cap-C3 could be caused by an impaired ability of injured cells to metabolize and remove complement proteins. Further studies need to be carried out to verify the speculation. Early treatment targeting complement might help slow the rate of complement activation in pediatric patients.

According to statistics, only 50% of patients with primary FSGS are successfully in remission after receiving IA. About 50% of individuals who fail to achieve remission will finally reach ESRD within 5–10 years (1, 15). New therapeutic strategies must be evaluated to improve survival. Therapy targeting complement, such as eculizumab, might be helpful therapies for children with primary FSGS and Cap-C3 deposition. These suggestions must be investigated in large, randomized, controlled clinical trials.

The activation of the complement system could be detected in the circulation and urine of primary FSGS patients (15), which prompts the clinical utility of some complement components as biomarkers for the clinical evaluation of kidney injury and outcomes. A recent study showed that the urine proteome panel, such as C4b, C9 and complement factor B and I, reflects damage to podocytes of patients with primary FSGS (26). The team of Zhang et al. further examined the plasma and urinary complement profile of seventy patients with primary FSGS and found that urinary C3a level was associated with poor renal outcomes of primary FSGS patients in the univariate Cox analysis (27). However, the multivariate Cox analysis showed that urinary C3a level was not an independent risk factor for poor renal outcome, while the urinary Bb level was independently associated with poor prognosis (27). But the urinary Bb level was positively correlated with the C3a level (27). Therefore, the interaction between urinary C3a and Bb levels might cause the insignificance of urinary C3a in the multivariate model. A larger cohort and a more rational model are needed to verify the value of urinary complement components as noninvasive biomolecules whose association and evaluation have the highest chances of correctly and indeed predicting the renal outcome in pediatric primary FSGS.

The strengths of our study are as follows. First, the sample size of our cohort was relatively large. Second, this study firstly revealed that Cap-C3 is a valuable predictor of the prognosis of pediatric primary FSGS. The limitations of this study are as follows. First, this is a retrospective study, which is possibly causing biases by construction. Second, despite excluding the clear secondary forms of FSGS, such as Alport syndrome, 187 (70.8%) patients have not yet taken the genetic test. Thus, a part of patients with genetic FSGS might not be recognized in this study.

## Conclusion

In summary, this study found that Cap-C3 deposition is an independent risk factor for poor renal survival in pediatric primary FSGS. These findings agree with the potential theory that complement activation is involved in the progression of FSGS. Nevertheless, the cause of complement activation in some patients with FSGS is yet unclear, necessitating further research.

## Data Availability

The raw data supporting the conclusions of this article will be made available by the authors, without undue reservation.
